# Popcorn: prediction of short coding and noncoding genomic sequences in prokaryotes

**DOI:** 10.1093/bioinformatics/btaf250

**Published:** 2025-04-25

**Authors:** Alison Kyrouz, Lian Liu, Lixin Qin, Brian Tjaden

**Affiliations:** Department of Computer Science, Wellesley College, Wellesley, MA 02481, United States; Department of Computer Science, Wellesley College, Wellesley, MA 02481, United States; Department of Computer Science, Wellesley College, Wellesley, MA 02481, United States; Department of Computer Science, Wellesley College, Wellesley, MA 02481, United States

## Abstract

**Summary:**

The most challenging prokaryotic genes to identify often correspond to short ORFs (sORFs) encoding small proteins or to noncoding RNAs. RNA-seq experiments commonly evince small transcripts that do not correspond to annotated genes and are candidates for novel coding sORFs or small regulatory RNAs, but it can be difficult to accurately assess whether the numerous small transcripts are coding or not. We present Popcorn (PrOkaryotic Prediction of Coding OR Noncoding), a novel machine learning method for determining whether prokaryotic sequences are coding or noncoding. We find that Popcorn is effective in distinguishing coding from noncoding sequences, including coding sORFs and noncoding RNAs.

**Availability and implementation:**

Freely available for use on the web at https://cs.wellesley.edu/∼btjaden/Popcorn. Source code available at https://github.com/btjaden/Popcorn and https://doi.org/10.5281/zenodo.15120075.

## 1 Introduction

Small proteins, also called microproteins or small peptides, are commonly defined as proteins containing up to 50 amino acids. The short ORFs (sORFs) encoding small proteins are generally overlooked by popular gene annotation systems owing to the challenges in accurately identifying short genes (Fuchs and Engelmann 2023). Yet, small proteins are prevalent in eukaryotes (Schlesinger and Elsasser 2022), prokaryotes (Duan *et al.* 2024), and phages (Fremin *et al.* 2022), and have important and varied functional roles, such as in regulation (Altieri *et al.* 2016), sporulation (Schmalisch *et al.* 2010), and as components of toxin-antitoxin systems (Sonika *et al.* 2023). Among prokaryotes, it has been observed that small proteins occur at a proportionally higher rate in archaea than in bacteria (Duan *et al.* 2024). In *Escherichia coli* (*E. coli*), perhaps the best studied prokaryote, more than 150 small proteins have been characterized and it is hypothesized that many more remain to be identified (Hemm *et al.* 2020, Gray *et al.* 2022).

Prokaryotic RNA-seq experiments often detect hundreds or even thousands of short transcripts that do not correspond to previously annotated genes (Sharma *et al.* 2010, Thomason *et al.* 2015). It remains a major open question as to what extent these transcripts correspond to previously uncharacterized genes, including new sRNAs (small noncoding regulatory RNAs) or protein encoding sORFs, or instead reflect noise and promiscuous transcription (Wade and Grainger 2014). While experimental approaches, such as ribosome profiling (Vazquez-Laslop *et al.* 2022) and mass spectrometry (Ahrens *et al.* 2022), can be effective in identifying small proteins, they do not necessarily scale with the pervasiveness of RNA-seq experiments and their detection of novel short transcripts. Thus, there is value in having computational methods, which can be more efficient than experimental methods, for determining whether novel transcripts observed from RNA-seq experiments are likely to represent protein encoding sORFs.

Owing to the importance of identifying which short transcripts are coding or not in the current age of abundant RNA-seq data, a number of computational tools have been developed for distinguishing coding sequences from noncoding sequences (Washietl *et al.* 2011, Zhu and Gribskov 2019, Zhang *et al.* 2021). The vast majority of tools for predicting protein encoding sORFs have been designed for eukaryotic data [reviewed in Schlesinger and Elsasser (2022)]. For prokaryotes, studies aimed at characterizing coding sORFs have focused primarily on well conserved families of small proteins. For instance, Sberro *et al.* (2019) search for instances of small proteins from conserved families in the human microbiome, and GMSC-mapper searches sequences against its large catalog, GMSC, of microbial sORF families in order to identify new members of the families (Duan *et al.* 2024). One of the limitations of these methods is that they are ineffective for sORFs that are not well conserved, which is common among these short sequences.

Here, we present a new method, Popcorn (PrOkaryotic Prediction of Coding OR Noncoding), for predicting whether prokaryotic sequences are coding or noncoding. Our method makes a number of contributions. First, it uses a neural network trained exclusively on prokaryotic data, including a large number of sORFs coding for small proteins (as examples of coding sequences) and a large number of sRNAs (as examples of noncoding sequences). For RNA-seq experiments that evince small transcripts that do not correspond to annotated genes, these training data enable Popcorn to effectively distinguish coding and noncoding sequences for common small gene families in prokaryotes. Second, Popcorn does not require sequences to be conserved, which is advantageous since many small genes both coding and noncoding are not well conserved. Third, for effective prediction, Popcorn does not require that a sequence begin and end precisely at transcription or translation initiation and termination points. A number of tools for eukaryotic data are trained and tested on sequences corresponding to coding ORFs, i.e. the translated portion of a gene beginning at a start codon and ending at a stop codon. In practice, transcripts observed from bulk RNA-seq experiments are not so precise and may represent only part of a gene owing, e.g. to processing or degradation, or may extend beyond the coding sequence because they include UTRs (untranslated regions). To address this issue, Popcorn was trained both on truncated sequences and on sequences extended upstream and downstream of a gene boundary. Fourth, to enable ease of use and reproducibility, Popcorn is available via a webserver at https://cs.wellesley.edu/∼btjaden/Popcorn.

## 2 Results

**Figure 1.**
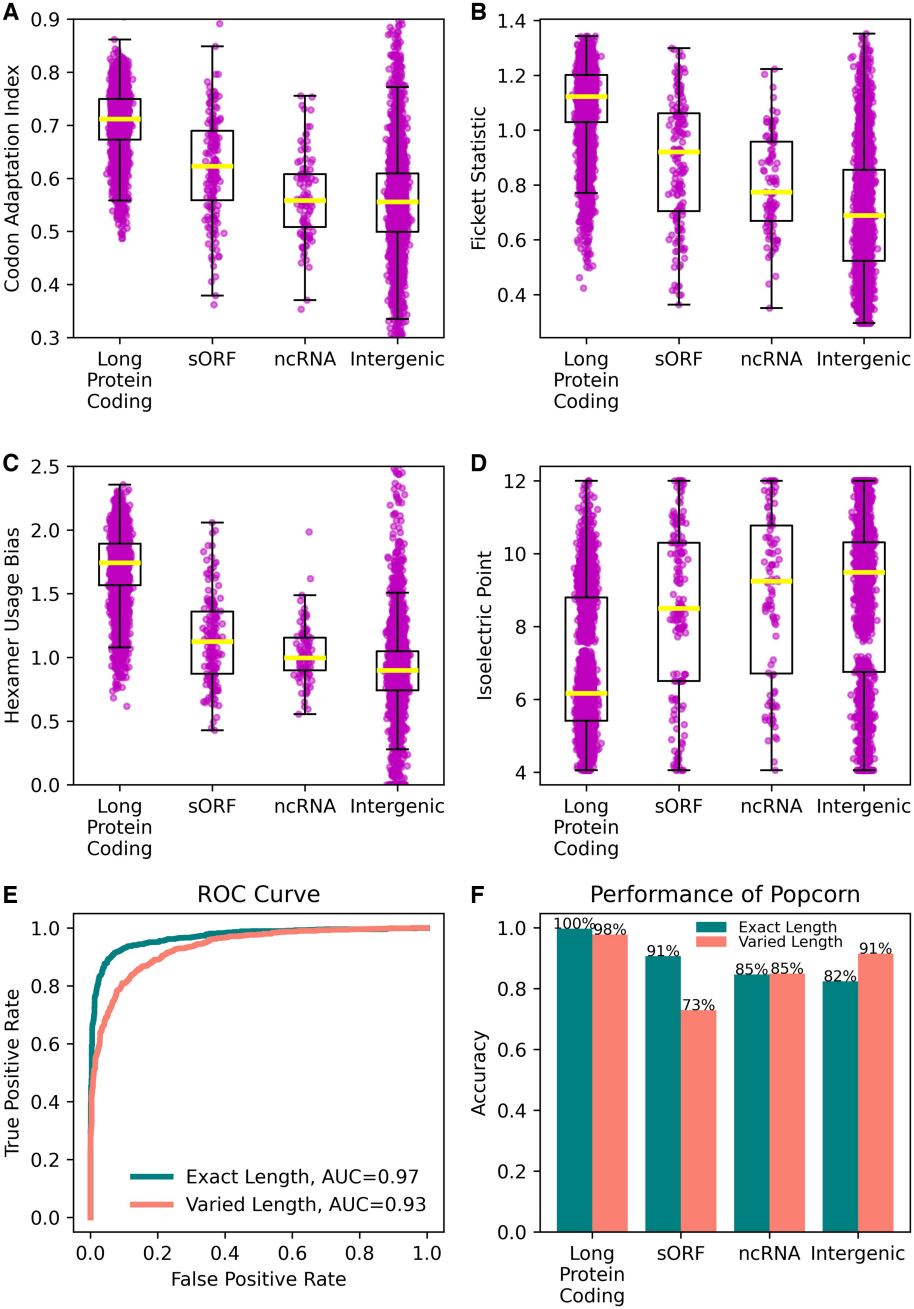
For four of the features, (A) Codon Adaptation Index, (B) Fickett Statistic, (C) Hexamer Usage Bias, and (D) Isoelectric Point, the distribution of the feature’s values is shown for each of four types of genomic sequences: long protein coding (coding for proteins with >50 amino acids), sORFs (coding for proteins with no >50 amino acids), ncRNAs (noncoding RNA genes, e.g. sRNAs, that do not correspond to “housekeeping” RNAs such as rRNAs and tRNAs), and noncoding intergenic sequences. Overlayed on each distribution is a box and whisker plot, where the horizontal line indicates the median of the distribution, the top and bottom of the box indicate the first and third quartiles of the distribution, and the whiskers extend from the box by 1.5 times the inter-quartile range. (E) the receiver operating characteristic (ROC) curve illustrating the performance of the trained neural network model on the testing data, on both exact length sequences and varied length sequences. (F) the performance of the neural network model on testing data as measured by accuracy on both exact length sequences and varied length sequences for four types of genomic sequences: long protein coding, sORFs, ncRNAs, and intergenic sequences.

We gathered a large set of prokaryotic genomic sequences corresponding to small genes, such as protein coding sORFs and ncRNAs, and we examined a variety of sequence features that may have predictive power in distinguishing the sequences as coding or noncoding (Supplementary Material). We investigated the distributions of features for four different types of sequences: long protein coding, sORFs, ncRNAs, and intergenic. The four types of sequences reflect, respectively, long coding sequences, short coding sequences, short noncoding sequences, and long noncoding sequences. We observed that the features indeed had different distributions for different types of sequences (Fig. 1A–D). For example, we found that the codon adaptation index (Fig. 1A), Fickett statistic (Fig. 1B), and hexamer usage bias (Fig. 1C) were higher for coding sequences (long protein coding and sORFs) and lower for noncoding sequences (ncRNAs and intergenic), whereas the isoelectric point (Fig. 1D) was lower for coding sequences (long protein coding and sORFs) and higher for noncoding sequences (ncRNAs and intergenic). Thus, these features contain some discriminatory properties and might reasonably be combined to discern coding from noncoding sequences.

Motivated by these results, we trained a neural network that integrates the different features (Supplementary Material). We then explored to what extent each feature contributes to the neural network’s output (Tjaden and Tjaden 2023). We calculated Shapley values to explain the neural network’s reliance on each feature (Lundberg and Lee 2017). We observed that some features, like the Fickett statistic, contribute more to the neural network’s inference whereas other features, such as the GC content and isoelectric point, contribute much less (Supplementary Fig. S1).

We evaluated the performance of our trained neural network model on testing data from prokaryotic genomes whose genus was not represented in the training data. We considered the AUC (area under the ROC curve) for both exact length sequences and varied length sequences that may be truncated and/or contain sequence upstream and downstream of the gene. In general, the model showed strong performance in characterizing coding and noncoding sequences, with AUCs of 0.97 and 0.93 for exact length sequences and varied length sequences, respectively (Fig. 1E). We also examined how the model performed on different types of sequences, namely on short (ncRNA and sORF) and long (intergenic and long protein coding) noncoding and coding sequences. For exact length sequences, the model correctly identified the sequence as coding or noncoding between 82% (intergenic) and 100% (long protein coding) of the time (Fig. 1F). For varied length sequences, the model correctly identified the sequence as coding or noncoding between 73% (sORF) and 98% (long protein coding) of the time (Fig. 1F).

The somewhat lower performance (73%) on varied length sORF sequences suggests that, for these sequences, the model identifies them as coding more accurately when the sequence more precisely reflects the sORF as opposed to noncoding regions upstream/downstream or truncated portions of the sORF. To test this hypothesis, we explored how the performance of the model changes for sequences that include different length regions upstream/downstream or that are truncated by different amounts. For noncoding sequences, using the precise boundary of the sequence has little effect on identifying the sequence as noncoding (Supplementary Fig. S2). However, for coding sequences, the model performs best when the entire coding region is included and the performance drops as smaller portions of the coding sequence are considered (Supplementary Fig. S2). It is perhaps unsurprising that the performance of the model is compromised most for sORFs when the integrity of the sequence length is varied since these are the shortest types of sequences, averaging 127 nucleotides in length.

Arguably the most challenging family of genes to characterize as coding or noncoding are dual-function sRNAs that act both as base-pairing regulatory RNAs and as peptide encoding mRNAs. A recent review defines 11 such dual-function sRNAs that have been identified throughout prokaryotes to date (Schnoor *et al.* 2024). To assess the model’s ability to characterize these difficult genes, for each of the 11 dual-function sRNAs we tested the model on the entire RNA sequence as well as on the peptide encoding portion of the sequence. We found that the model identifies all 11 peptide encoding regions as coding regions and identifies 7 of the 11 complete RNA sequences as noncoding (Supplementary Fig. S3), suggesting that the model performs well but not perfectly on these challenging genes.

## 3 Discussion

Popcorn is a tool for determining whether prokaryotic genomic sequences are protein coding or noncoding. Popcorn uses a neural network trained on a large set of prokaryotic sequences, including short coding and noncoding genes. A common use case for Popcorn arises following an RNA-seq experiment where small transcripts are observed that do not correspond to annotated genes. Popcorn can distinguish protein coding sORFs from other transcripts such as noncoding RNAs, which are some of the most challenging prokaryotic genes to identify owing to their small size and, often, their lack of conservation in other species. Popcorn is available to use via a user-friendly web interface at https://cs.wellesley.edu/∼btjaden/Popcorn.

## Supplementary Material

btaf250_Supplementary_Data

## Data Availability

Source code is available at https://github.com/btjaden/Popcorn and https://doi.org/10.5281/zenodo.15120075.

## References

[btaf250-B1] Ahrens CH , Wade JT, Champion MM et al A practical guide to small protein discovery and characterization using mass spectrometry. J Bacteriol 2022;204:e0035321. 34748388 10.1128/jb.00353-21 PMC8765459

[btaf250-B2] Altieri AS , Ladner JE, Li Z et al A small protein inhibits proliferating cell nuclear antigen by breaking the DNA clamp. Nucleic Acids Res 2016;44:6232–41. 27141962 10.1093/nar/gkw351 PMC5181682

[btaf250-B3] Duan Y , Santos-Júnior CD, Schmidt TS et al A catalog of small proteins from the global microbiome. Nat Commun 2024;15:7563. 39214983 10.1038/s41467-024-51894-6 PMC11364881

[btaf250-B4] Fremin BJ , Bhatt AS, Kyrpides NC et al; Global Phage Small Open Reading Frame (GP-SmORF) Consortium. Thousands of small, novel genes predicted in global phage genomes. Cell Rep 2022;39:110984. 35732113 10.1016/j.celrep.2022.110984 PMC9254267

[btaf250-B5] Fuchs S , Engelmann S. Small proteins in bacteria—big challenges in prediction and identification. Proteomics, 2023;23:e2200421. 37609810 10.1002/pmic.202200421

[btaf250-B6] Gray T , Storz G, Papenfort K. Small proteins; big questions. J Bacteriol 2022;204:e0034121. 34309401 10.1128/JB.00341-21 PMC8765408

[btaf250-B7] Hemm MR , Weaver J, Storz G. Escherichia coli small proteome. EcoSal Plus 2020;9:10–1128. 10.1128/ecosalplus.esp-0031-2019 PMC7212919 32385980

[btaf250-B8] Lundberg SM , Lee SI. A unified approach to interpreting model predictions. Adv Neural Inform Process Syst 2017;30:4765–74.

[btaf250-B9] Sberro H , Fremin BJ, Zlitni S et al Large-scale analyses of human microbiomes reveal thousands of small, novel genes. Cell 2019;178:1245–59 e14. 31402174 10.1016/j.cell.2019.07.016 PMC6764417

[btaf250-B10] Schlesinger D , Elsasser SJ. Revisiting sORFs: overcoming challenges to identify and characterize functional microproteins. FEBS J 2022;289:53–74. 33595896 10.1111/febs.15769

[btaf250-B11] Schmalisch M , Maiques E, Nikolov L et al Small genes under sporulation control in the *Bacillus subtilis* genome. J Bacteriol 2010;192:5402–12. 20709900 10.1128/JB.00534-10 PMC2950494

[btaf250-B12] Schnoor SB , Neubauer P, Gimpel M. Recent insights into the world of dual-function bacterial sRNAs. Wiley Interdiscip Rev RNA 2024;15:e1824. 10.1002/wrna.1824 38039556

[btaf250-B13] Sharma CM , Hoffmann S, Darfeuille F et al The primary transcriptome of the major human pathogen *Helicobacter pylori*. Nature 2010;464:250–5. 20164839 10.1038/nature08756

[btaf250-B14] Sonika S , Singh S, Mishra S et al Toxin-antitoxin systems in bacterial pathogenesis. Heliyon 2023;9:e14220. 37101643 10.1016/j.heliyon.2023.e14220 PMC10123168

[btaf250-B15] Thomason MK , Bischler T, Eisenbart SK et al Global transcriptional start site mapping using differential RNA sequencing reveals novel antisense RNAs in *Escherichia coli*. J Bacteriol 2015;197:18–28. 25266388 10.1128/JB.02096-14 PMC4288677

[btaf250-B16] Tjaden J , Tjaden B. MLpronto: a tool for democratizing machine learning. PLoS One 2023;18:e0294924. 38032968 10.1371/journal.pone.0294924 PMC10688639

[btaf250-B17] Vazquez-Laslop N , Sharma CM, Mankin A et al Identifying small open reading frames in prokaryotes with ribosome profiling. J Bacteriol 2022;204:e0029421. 34339296 10.1128/JB.00294-21 PMC8765392

[btaf250-B18] Wade JT , Grainger DC. Pervasive transcription: illuminating the dark matter of bacterial transcriptomes. Nat Rev Microbiol 2014;12:647–53. 25069631 10.1038/nrmicro3316

[btaf250-B19] Washietl S , Findeiss S, Müller SA et al RNAcode: robust discrimination of coding and noncoding regions in comparative sequence data. RNA 2011;17:578–94. 21357752 10.1261/rna.2536111 PMC3062170

[btaf250-B20] Zhang Y , Jia C, Fullwood MJ et al DeepCPP: a deep neural network based on nucleotide bias information and minimum distribution similarity feature selection for RNA coding potential prediction. Brief Bioinform 2021;22:2073–84. 32227075 10.1093/bib/bbaa039

[btaf250-B21] Zhu M , Gribskov M. MiPepid: microPeptide identification tool using machine learning. BMC Bioinformatics 2019;20:559. 31703551 10.1186/s12859-019-3033-9 PMC6842143

