# Supplementary material for: Popcorn: prediction of short coding and noncoding genomic sequences in prokaryotes
Source: Bioinformatics. 2025 Apr 25;41(5):btaf250. doi: 10.1093/bioinformatics/btaf250 (PMC12054974; doi:10.1093/bioinformatics/btaf250)
Supplement: btaf250_Supplementary_Data [file btaf250_supplementary_data.pdf]

Supplementary Material

Popcorn: prediction of short coding and noncoding genomic sequences in prokaryotes

Data

For the approximately 5,000 bacterial and archaeal genomes labeled as "reference" or "representative" in RefSeq [1], we considered all annotated genes and intergenic regions. From the annotated genes, we extracted information on the 180,918 sORFs (coding sequences that correspond to proteins with no more than 50 amino acids) and the 4,442 ncRNAs (noncoding RNAs that do not correspond to "housekeeping" RNAs such as rRNAs and tRNAs). For *testing* data, we used sequences from the 15 "RefSeq selected reference [2]" bacterial genomes (*Acinetobacter pittii* PHEA-2, *Bacillus subtilis* subsp. subtilis str. 168, *Campylobacter jejuni* subsp. jejuni NCTC 11168 = ATCC 700819, *Caulobacter vibrioides* NA1000, *Chlamydia trachomatis* D/UW-3/CX, *Coxiella burnetii* RSA 493, *Escherichia coli* O157:H7 str. Sakai, *Escherichia coli* str. K-12 substr. MG1655, *Klebsiella pneumoniae* subsp. pneumoniae HS11286, *Listeria monocytogenes* EGD-e, *Mycobacterium tuberculosis* H37Rv, *Pseudomonas aeruginosa* PAO1, *Salmonella enterica* subsp. enterica serovar Typhimurium str. LT2, *Shigella flexneri* 2a str. 301, *Staphylococcus aureus* subsp. aureus NCTC 8325) and 3 archaeal sets of genomes (*Haloferax*, *Methanobacterium*, *Thermococcus*). To ensure no data leakage between *training* and *testing* data, we removed any sequences from the *training* data that were from the same genus as any of the *testing* data. Owing to the class imbalance, i.e., 98% of this small gene data represents coding rather than noncoding sequences, we randomly undersampled the majority class to achieve balanced classes.

In order to investigate more varied gene sequences than exact gene sequences (from the precise start of the gene to the precise stop of the gene), we considered different starting and stopping

points for each gene sequence. The motivation, here, is that bulk RNA-seq experiments don't always evince the precise extent of a gene, sometimes capturing only part of a transcript and sometimes capturing transcription upstream (or downstream) of the start (or end) of a coding sequence. Thus, to determine new starting and stopping points for each sequence, we sampled uniformly at random a window of 50 nucleotides centered at the start and centered at the stop of the gene, i.e., we added up to 25 nucleotides upstream of the gene start or truncated up to the first 25 nucleotides of the gene, and we added up to 25 nucleotides downstream of the gene stop or truncated up to the final 25 nucleotides of the gene.

Features

For each sequence, a variety of features are calculated that may help indicate whether the sequence is likely to be coding or noncoding. First, the longest ORF within the sequence is identified and the ORF coverage is determined as the percentage of the entire sequence represented by the longest ORF. For both of the entire sequence and the longest ORF within the sequence, we calculate how much the GC content differs from the genome's mean GC content, the codon adaptation index [3], Fickett TestCode statistic [4], hexamer usage bias [5, 6], and isoelectric point [7]. Feature values are scaled so that each feature has a mean of zero and a standard deviation of one.

Machine Learning

To understand the extent to which the features can be used to distinguish coding from noncoding sequences, we trained a neural network, i.e., a multi-layer fully-connected perceptron. We evaluated a range of hyperparameters for the neural network and its architecture based on their performance on validation data, tuning the neural network to optimize its validation performance. The tuned neural network consists of four layers, an input layer, two hidden layers, and an output layer. The two hidden layers contain 20 and 5 units, respectively, employing ReLU activation functions. The output layer uses a sigmoid activation function for binary classification. For optimizing weight parameters, the Adam method for stochastic gradient descent optimization was used for 1,000 epochs with the L2 regularization parameter set to 0.1. Altogether, the neural network contains 371 trainable parameters.

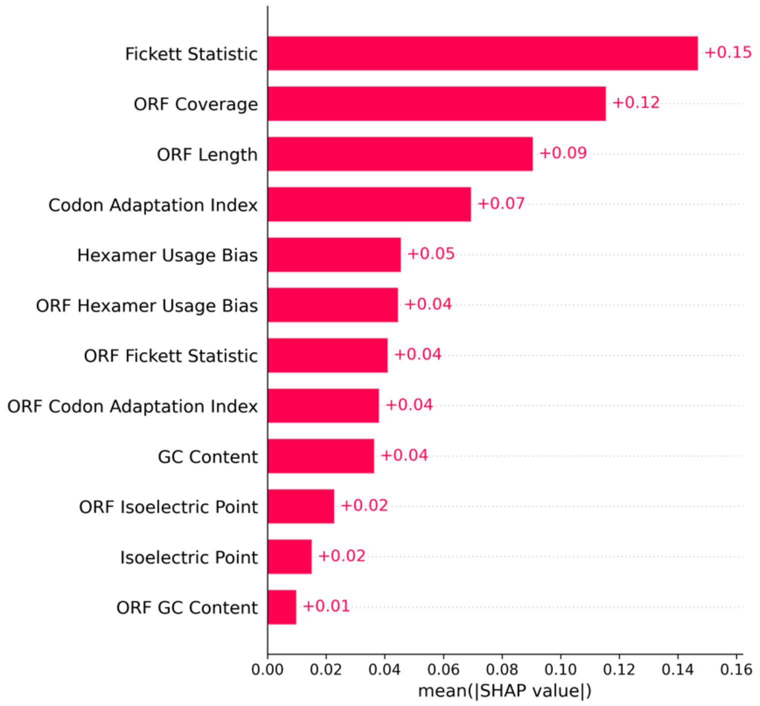

Supplementary Figure 1. The mean Shapley value for each of the 12 features is shown. A larger Shapley value indicates a feature enables greater explanation of the neural network model.

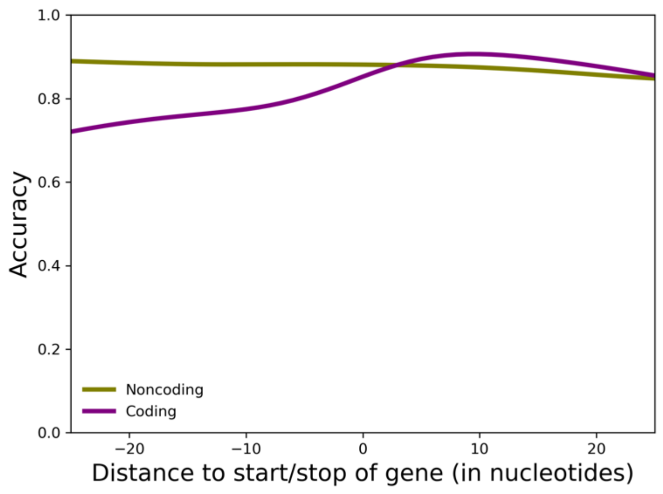

Supplementary Figure 2. The accuracy of the neural network model is illustrated for both noncoding and coding sequences as the length of the sequences are perturbed. Along the horizontal axis, a value of 0 corresponds to exact length gene sequences, a value of -25 corresponds to truncating 25 nucleotides from both the start and end of the sequence, and a value of 25 corresponds to including at the start of the sequence 25 nucleotides upstream of the start of the gene as well as including at the end of the sequence 25 nucleotides downstream of the end of the gene sequence.

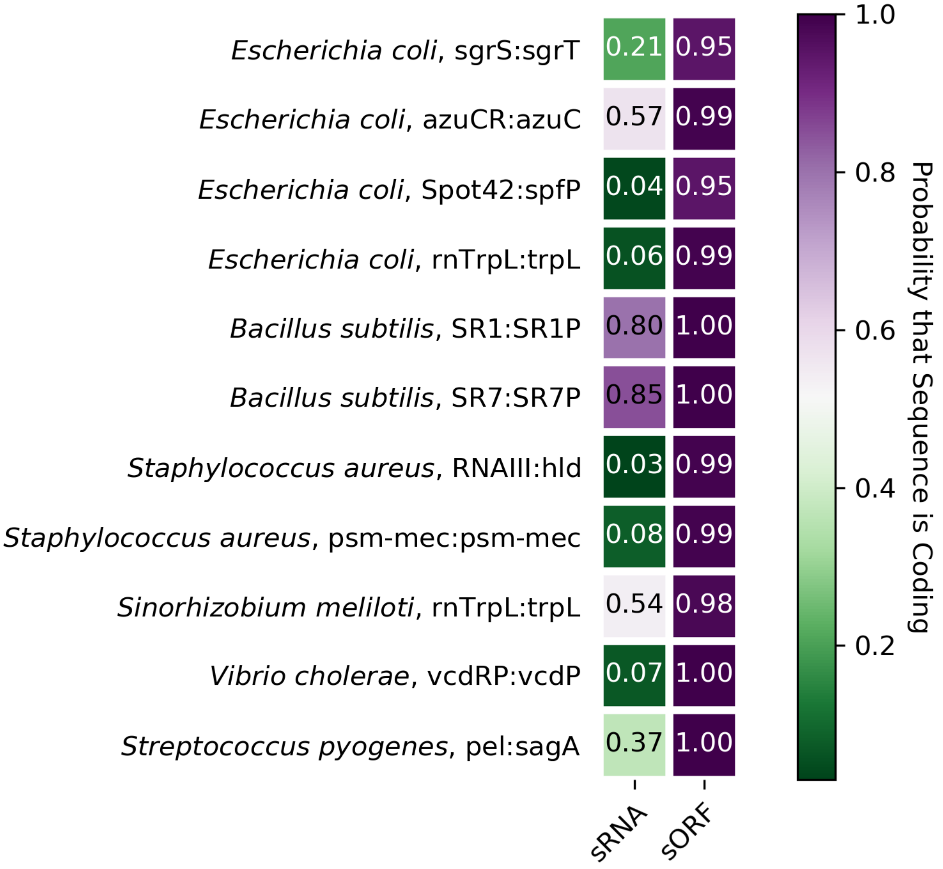

Supplementary Figure 3. For each of 11 dual-function sRNAs, two sequences were considered: the entire RNA sequence corresponding to the base-pairing regulatory RNA (sRNA column) and the portion of the sequence corresponding to a peptide encoding sORF (sORF column). The probability that each sequence is coding, as determined by the neural network model, is shown.

References

1. Li, W., et al., *RefSeq: expanding the Prokaryotic Genome Annotation Pipeline reach with protein family model curation.* Nucleic Acids Res, 2021. **49**(D1): p. D1020-D1028. PMC7779008
2. *Prokaryotic RefSeq Genomes*. Available from: https://www.ncbi.nlm.nih.gov/refseq/about/prokaryotes/#reference_genomes.
3. Sharp, P.M. and W.H. Li, *The codon Adaptation Index--a measure of directional synonymous codon usage bias, and its potential applications.* Nucleic Acids Res, 1987. **15**(3): p. 1281-95. PMC340524
4. Fickett, J.W., *Recognition of protein coding regions in DNA sequences.* Nucleic Acids Res, 1982. **10**(17): p. 5303-18. PMC320873
5. Fickett, J.W. and C.S. Tung, *Assessment of protein coding measures.* Nucleic Acids Res, 1992. **20**(24): p. 6441-50. PMC334555
6. Wang, L., et al., *CPAT: Coding-Potential Assessment Tool using an alignment-free logistic regression model.* Nucleic Acids Res, 2013. **41**(6): p. e74. PMC3616698
7. Bjellqvist, B., et al., *The focusing positions of polypeptides in immobilized pH gradients can be predicted from their amino acid sequences.* Electrophoresis, 1993. **14**(10): p. 1023-31.
